# Maternal feeding practices and fussy eating in toddlerhood: a discordant twin analysis

**DOI:** 10.1186/s12966-016-0408-4

**Published:** 2016-07-13

**Authors:** Holly A. Harris, Alison Fildes, Kimberley M. Mallan, Clare H. Llewellyn

**Affiliations:** Centre for Children’s Health Research, Queensland University of Technology, South Brisbane, 4101 Australia; School of Exercise and Nutrition Sciences, Queensland University of Technology, Kelvin Grove, Brisbane, 4059 Australia; Health Behaviour Research Centre, University College London, London, WC1E 6BT UK; School of Psychology, Australian Catholic University, Brisbane, 4014 Australia; University College of London, Gower Street, London, WC1E 6BT UK

**Keywords:** Food fussiness, Children, Feeding practices, Twin study, Eating behaviour, Diet

## Abstract

**Background:**

Parental feeding practices are thought to play a causal role in shaping a child’s fussiness; however, a child-responsive model suggests that feeding practices may develop in response to a child’s emerging appetitive characteristics. We used a novel twin study design to test the hypothesis that mothers vary their feeding practices for twin children who differ in their ‘food fussiness’, in support of a child-responsive model.

**Methods:**

Participants were mothers and their 16 month old twin children (*n* = 2026) from Gemini, a British twin birth cohort of children born in 2007. Standardized psychometric measures of maternal ‘pressure to eat’, ‘restriction’ and ‘instrumental feeding’, as well as child ‘food fussiness’, were completed by mothers. Within-family analyses examined if twin-pair differences in ‘food fussiness’ were associated with differences in feeding practices using linear regression models. In a subset of twins (*n* = 247 pairs) who were the most discordant (highest quartile) on ‘food fussiness’ (difference score ≥ .50), Paired Samples *T*-test were used to explore the magnitude of differences in feeding practices between twins. Between-family analyses used Complex Samples General Linear Models to examine associations between feeding practices and ‘food fussiness’.

**Results:**

Within-pair differences in ‘food fussiness’ were associated with differential ‘pressure to eat’ and ‘instrumental feeding’ (*p*s < .001), but not with ‘restriction’. In the subset of twins most discordant on ‘food fussiness’, mothers used more pressure (*p* < .001) and food rewards (*p* < .05) with the fussier twin. Between-family analyses indicated that ‘pressure to eat’ and ‘instrumental feeding’ were positively associated with ‘food fussiness’, while ‘restriction’ was negatively associated with ‘food fussiness’ (*p*s < .001).

**Conclusions:**

Mothers appear to subtly adjust their feeding practices according to their perceptions of their toddler’s emerging fussy eating behavior. Specifically, the fussier toddler is pressured more than their less fussy co-twin, and is more likely to be offered food rewards. Guiding parents on how to respond to fussy eating may be an important aspect of promoting feeding practices that encourage food acceptance.

**Electronic supplementary material:**

The online version of this article (doi:10.1186/s12966-016-0408-4) contains supplementary material, which is available to authorized users.

## Background

“Fussy” or “picky” eating is the tendency to reject a large proportion of new or familiar foods, often due to texture or flavor, and is associated with reduced dietary variety, particularly for nutrient-dense foods conducive to long-term health [1, 2]. The emergence of fussy eating behavior during toddlerhood is a normal part of child development [3], and prevalence has been reported to increase from 14 to 50 % between 4 and 24 months of age [4]. As early behaviors related to food rejection are largely heritable [5, 6], a conceivable mechanism of parent–child feeding interactions may be that children are driving their caregiver’s feeding practices through the expression of their genetically-influenced fussy eating traits – so-called ‘gene-environment correlation’. However, research to date has assumed that feeding practices have a causal influence on the development of fussy eating [7–9].

Highly controlling “non-responsive” parental feeding practices have been cross-sectionally associated with greater fussy eating in a number of studies [10–13], but it is not possible to determine the direction of effects from these findings. However, prospective studies have supported the idea that parents adapt their feeding practices based on their child’s various characteristics, suggesting a child-responsive model [14–17]. From a child-responsive perspective, we may speculate that past findings from cross-sectional studies [10–13] partly reflect parents of fussier eaters using practices such as pressuring the child to eat healthy foods, using the child’s favorite foods as a bribe to get them to eat disliked foods, or restricting the child’s access to unhealthy foods in an attempt to encourage their child to eat a wider variety of foods, or to increase their intake of healthier foods. Although, longitudinal evidence with cross-lagged models to disentangle the relationship between fussy eating and feeding practices is lacking.

Twin and sibling designs provide another angle through which parents’ response to differences in child characteristics may be studied; and this approach also controls for potentially confounding environmental influences that are shared by two twins or siblings in a family (e.g. parental eating behavior and weight, modelling of eating behavior by parents or other siblings in the family, socioeconomic status, etc.). The hypothesis is that a parent will only use different parenting or feeding strategies with two twins (or siblings), if he or she is responding to different behaviors expressed by each child. A sibling study has shown that parents use more non-responsive feeding practices for the fussier sibling [18]. While, a recent study of 4- to 7-year old twins (*n* = 64 pairs) showed that mothers reported less restrictive feeding practices for twins who compensated for caloric intake in a test meal, i.e. the twin who demonstrated the better ability to self-regulate their intake than their co-twin [19]. A twin study would also help to clarify the relationship between parental feeding practices and children’s fussy eating.

The aims of this study were to: i) identify whether parental feeding practices are responsive to differences in fussy eating traits at 16 months using a twin design; ii) determine the extent to which mothers’ feeding practices differ between twins in a subset identified as the most discordant (highest quartile) on ‘food fussiness’; iii) ascertain if the same associations between ‘food fussiness’ and feeding practices are observed between families, as within families. Three separate analyses were conducted in a large sample of twins to address each aim. Firstly, associations between twins’ fussy eating behavior and maternal feeding practices were examined in the full sample of twins. Secondly, we analyzed a subsample of the twins who were notably different in fussy eating behaviors, to provide additional information on the extent to which feeding practices differed within certain families. Finally, we examined associations between fussy eating and feeding practices between families, while controlling for key maternal and child covariates. This analysis allowed us to identify shared environmental confounders in the relationship between fussy eating and parental feeding practices that are controlled for in the within-family analysis. Together, these analyses provided a complete picture of this feeding relationship within- and between-families. Within-families, we hypothesized that: (aim i) the greater the difference in fussiness between twin pairs, the greater the difference in mothers’ use of ‘pressure to eat’, ‘instrumental feeding’ and ‘restriction’ between pairs; and that (aim ii) within pairs discordant for fussiness, the mother would use more ‘pressure to eat’, more ‘restriction’, and more ‘instrumental feeding’ with the fussier twin. Between families (aim iii) we hypothesized that ‘pressure to eat’, ‘instrumental feeding’ and ‘restriction’ would be positively associated with child ‘food fussiness’.

## Methods

### Sample

Participants were mothers of twins from the Gemini birth cohort; a study conducted in the United Kingdom assessing genetic and environmental influences on child diet, activity and early growth. Recruitment methods have been reported elsewhere [20]. Briefly, families with twins born in England and Wales between March and December 2007 were invited to participate (*n* = 6754) by the Office of National Statistics. Ethical approval was granted by the University College London Committee for the Ethics of non-National Health Service Human Research. Of the families who agreed to be contacted by the Gemini research team (*n* = 3435), 2402 families completed the baseline questionnaire (70 % response rate). Twins in the sample are comparable to national twin statistics on sex, gestational age and birth weight. However, as expected with cohort studies, the sample is over-representative of parents of white ethnicity, with a level of high educational qualifications and occupational status in comparison to the UK population [21]. Data in the current study are from the baseline and first follow-up questionnaires, completed when the children were on average 8 months and 16 months old, respectively. At 16 months, 1931 families remained active in Gemini (*n* = 3862 children) (19.6 % attrition). Participants in the current study were 1013 families with complete data on all variables in the analysis. Of the 1013 twin pairs, 344 (34.0 %) pairs were monozygotic, 654 (65.5 %) pairs were dizygotic and 15 (1.5 %) pairs were of unknown zygosity.

### Sociodemographic and anthropometric measures

Sociodemographic information was collected at baseline. Mothers were asked to report their age at delivery (years), highest level of educational qualification obtained, ethnicity and their current height (cm) and weight (kg), which were used to calculate Body Mass Index (BMI) (kg/m^2^). There were 19 mothers with missing BMI values for whom the mean BMI of the total sample at baseline (25.12 kg/m^2^) was imputed. Educational qualifications were categorised as “low” (no qualification/ basic high-school), “intermediate” (vocational/ advanced high-school) and “high” (university). Ethnicity was dichotomised into “white British” and “non-white British”. Mothers reported the gestational age of the twins at delivery (weeks) and each child’s sex. The feeding method used in the infant’s first three months of life was indicated by the mother from the following options: “exclusive breast feeding”, “mostly breast, some bottle”, “equal breast and bottle feeding”, “mostly bottle feeding, some breastfeeding”, “almost entirely bottle feeding” and “entirely bottle”. Responses were categorised as: “entirely, mostly or equally breastfed for 3 months” (Mostly breastfed) and “entirely or mostly bottle-fed for 3 months” (Mostly bottle-fed).

Weights were largely based on measurements made by health professionals (96.4 % of data), recorded in the child’s personal health record. When weights recorded by health professionals were unavailable, parents were asked to measure and record their children’s weights themselves. Weights at birth and 16 months were converted into weight standard deviation scores (SDS) using UK 1990 reference data, which account for the child’s age (at the time of measurement), sex and gestational age [22]. Weight SDS of 0 indicates the child had the mean weight for a child of the same age, sex and gestational age in 1990; a weight SDS greater than 0 indicates a higher weight relative to the 1990 mean reference value; a value less 0 indicates a lower weight, relative to the 1990 mean reference value.

### Measures

#### Parental feeding practices

‘Instrumental feeding’, ‘pressure to eat’ and ‘restriction’ were measured using scales from the Parental Feeding Style Questionnaire (PFSQ) [23], the Child Feeding Questionnaire (CFQ) [12] and a questionnaire developed for a previous study (Poppets) [24], respectively. Where necessary, items were modified to be age-appropriate and piloted intensively in a small sample of mothers with 18-month old toddlers (*n* = 12) (details of the final items included in each scale are summarised in Additional file 1). The ‘instrumental feeding’ scale from the PFSQ measures parents’ use of food as a contingency for healthy food consumption or good behaviour (3 items; α = 0.54). The ‘pressure to eat’ scale from the CFQ was used to measure parents’ attempts to coerce the child to eat more (5 items; α = 0.66). ‘Restriction’ was measured using a scale specifically designed for a previous study to measure parental tendency to limit the child’s access to, and portion sizes of, sugary and high fat foods (4 items; α =0.85). Mothers were asked to indicate how frequently these practices were used on a 5-point Likert scale, from “never” (1) to “always” (5) for ‘instrumental feeding’ and ‘pressure to eat’. ‘Restriction’ was measured on a 7-point Likert scale, and mothers were asked to indicate how firm they were with their limitations of foods from “not at all” (1) to “strictly” (7). Higher scores indicated more frequent use of the feeding practice. Where responses were missing for more than 2/3, 3/4, or 3/5 items, cases were excluded from the analysis.

### Food fussiness

Fussy eating was measured at 16 months using the ‘food fussiness’ scale from the Children’s Eating Behaviour Questionnaire (CEBQ) [25]. The CEBQ is a parent-report standardised psychometric measure of eight eating behaviors in children; it has good construct validity and test-retest reliability, and has been validated in samples of children aged one to five-years of age [26]. Mothers responded to 6 items that described a child’s fussiness regarding food (e.g. “My child is difficult to please with meals”) using a 5-point Likert scale from “never” (1) to “always” (5), with higher scores indicating greater fussiness (α = 0.87). Cases with responses missing on more than 4/6 items were excluded from the analysis.

### Data analysis

All statistical analyses were carried out using SPSS (version 20; IBM SPSS Statistics, IBM Corporation, Armonk, NY). Three specific analyses were conducted to address each aim. To address the first aim, within-family differences between twin pairs on ‘food fussiness’ and maternal feeding practices were calculated by subtracting scores for Twin 1 (first born) from Twin 2 (second born) using a similar method to that of Farrow and colleagues [18]. Differences in ‘food fussiness’ were correlated with differences in feeding practices within families using Pearson’s Correlation Coefficients. Within-pair ‘food fussiness’ difference was entered into a linear regression model (as the independent variable) to determine if this was related to within-pair differences in feeding practices (entered as the dependent variables, in separate models). The models controlled for differences between twins in: birth weight SDS; child weight SDS at 16 months (the weight measurement taken nearest to the time that the parents completed the questionnaire measures of their feeding styles, and their children’s food fussiness); early feeding method; and sex. Early feeding method was dummy coded such that a different feeding method between twins was the reference group and child sex was dummy coded such that opposite-sex twins was the reference group.

To address the second aim, Paired Samples T-tests were used to explore in more detail differences in feeding practices for a subset of twins who were distinctly different (discordant) in ‘food fussiness’. This analysis enabled us to explore significant differences in parental feeding practices between the ‘fussy twin’ and ‘less fussy twin’. ‘Discordant twins’ in the sample were defined as twin pairs who had ‘food fussiness’ difference scores that were greater than or equal to the upper quartile (75th percentile). The upper quartile of differences in ‘food fussiness’ between twin pairs was ≥ 0.50.

To address the third aim, between-family analyses were run to ascertain if the same patterns of associations that were observed within families were also observed between families. This analysis helps to identify potential confounders in the relationship between parental feeding and child ‘food fussiness’ that are shared between twin pairs and therefore controlled for in the within-family analysis. Complex Samples General Linear Models were used to assess the relationship between each feeding practice (entered as a dependent variable in separate models) and ‘food fussiness’ (entered as the independent variable in each model), controlling for potential confounders, and adjusting for clustering of the twins in families. Confounders were chosen a priori and included: maternal age at delivery; maternal education; maternal ethnicity; maternal BMI; gestational age; child age at questionnaire completion; birth weight SDS; child weight SDS at the time of measurement (16 months); and predominant early feeding method in the first 3 months of life.

## Results

The characteristics of the full Gemini sample at baseline and the study sample are shown in Table 1. Non-response analyses indicated that mothers included in the study sample were more likely to be older (Mean (SD) 33.6 (5.00) vs. 32.5 (5.28), *p* < .001), have a lower BMI (24.3 (4.84) kg/m^2^ vs. 25.3 (4.84) kg/m^2^, *p* = .009), be university educated (48.5 % vs. 41.9 % *p* < .001), and mostly breastfeed (*p* < .001) in comparison to mothers who did not provide complete data or were lost to follow-up. Children in the study sample were more likely to be lighter at birth (2.4 (.55) kg vs. 2.5 (.53) kg, *p* = .004) than children who had missing data or were lost to follow up, although there was no difference in birth weight SDS (*p* = .55).

**Table 1:** Characteristics of the full Gemini sample at baseline compared to the study sample

Child characteristics	Gemini sample at baseline (*n* = 4804 children)	Study sample (*n* = 2026 children)
	Mean (SD) or *n* (%)	Mean (SD) or *n* (%)
Gender (female)	2386 (49.7)	1058 (52.2)
Birth weight (kg)	**2.5 (.54)**	2.4 (.55)*
Birth weight SDS	−.56 (.95)	−.57 (.94)
Weight at 16 months (kg)	**–**	10.3 (1.25)
Weight SDS at 16 months	**–**	−.08 (1.08)
Gestational age (weeks)	36.2 (2.48)	36.1 (2.60)
Age questionnaire completion	–	15.8 (.90)
Early feeding method Mostly breastfed Mostly bottle-fed	**2017 (42.0)** 2755 (57.3)	946 (46.7)* 1080 (53.7)
Maternal characteristics		
Age at twins’ birth (years)	**33.0 (5.19)**	33.6 (5.00)*
BMI (kg/m^2^)	**25.1 (4.70)**	24.9 (4.49)*
Highest Level of Education Attained Low (No qualification/basic high-school) Intermediate (Vocational/Adv. high-school) High (University)	518 (21.6) 878 (36.6) **1006 (41.9)**	171 (16.9) 351 (34.6) 491 (48.5)*
Ethnicity White British Non-White British/Unknown	2089 (87.0) 313 (13.0)	896 (88.5) 117 (11.5)
Feeding practices and Food Fussiness, mean (SD)		
Food Fussiness^a^	–	2.18 (.71)
Instrumental Feeding^b^	–	1.33 (.46)
Pressure to Eat^c^	–	2.22 (.74)
Restriction^d^	–	5.21 (1.26)

Abbreviations: *Weight SDS:* Weight Standard Deviation Score, *kg:* kilograms, *BMI:* Body Mass Index

*p < .01; **p < .001

Subscales are from the ^a^CEBQ [25]; ^b^PFSQ [23]; ^c^CFQ [12]; and ^d^Poppets scale [24]

Of the families active at the 16 month follow-up, mothers included in the study sample (*n* = 1013) were more likely to be university educated (48.5 % vs. 41.4 %, *p* < .001), and mostly breastfeed (46.7 % vs. 42.6 %, *p* < .01) in comparison to mothers who were excluded due to incomplete data (*n* = 918). Children in the study sample (*n* = 2026) were more likely to be younger at the time of completion of the 16 month questionnaire (15.8 (.90) months vs. 15.9 (1.37) months, *p* < .01), and have a lower birthweight (2.4 (.55) kg vs. 2.5 (.52) kg, *p* < .001) than children who had incomplete data (*n* = 1836), although there was no difference in birth weight SDS (*p* = .18). There were no significant differences between child ‘food fussiness’ and any of the feeding practices, except mothers in the excluded group reported higher levels of instrumental feeding (1.39 (.48) vs. 1.33 (.46) of maximum score of 5, *p* < .01).

Descriptive statistics of the main variables of interest for Twin 1 and Twin 2 (first and second born, respectively) are reported in Table 2. Paired Samples T-tests indicated that there were no significant differences between first and second born twins for ‘food fussiness’ or any of the feeding practices. However, Twin 2 was more likely to have a lower weight SDS score at birth in comparison to Twin 1 (M ± SD = −.65 ± .98 vs. -.48 ± .88, t (1012) = 5.37, *p* < .001), and at 16-months of age (M ± SD = −.16 ± 1.09 vs. .01 ± 1.07, t(1012) = 5.48, *p* < .001). The number of twin pairs who had a difference score >0 (i.e. twin pairs who were different) for each of the independent variables (feeding practices) and the dependent variable (‘food fussiness’) are also reported in Table 2.

**Table 2:** Descriptive statistics for Twin 1 and Twin 2 for ‘food fussiness’ and feeding practices

	Twin 1 *n* = 1013	Twin 2 *n* = 1013	Number of pairs (%) with a difference score > 0
	Mean (SD)		
Food fussiness^a^	2.16 (.70)	2.19 (.72)	522 (51.5)
Instrumental feeding^b^	1.33 (.46)	1.33 (.46)	30 (3.0)
Pressure to eat^c^	2.21 (.73)	2.22 (.74)	205 (20.2)
Restriction^d^	5.21 (1.25)	5.20 (1.26)	19 (1.9)

Subscales are from the ^a^CEBQ [25]; ^b^PFSQ [23]; ^c^CFQ [12]; and ^d^Poppets scale [24]

### Within-pair analyses

Differences in ‘food fussiness’ between twins was significantly positively correlated with differences in ‘pressure to eat’ (*r* = .36, *p* < .001), such that there were larger differences between twins pairs for maternal ‘pressure to eat’, for twin pairs who differed more in their ‘food fussiness’. There was also a small but significant positive correlation between differences in ‘food fussiness’ and differences in ‘instrumental feeding’ (*r* = .15, *p* < .001), such that there were larger differences between twin pairs for maternal use of food as a reward, for twin pairs who differed more in their ‘food fussiness’. There was no association between twin pair differences in ‘restriction’ and ‘food fussiness’ (*p* = .30). Multiple linear regressions, adjusting for other differences between twin pairs, confirmed the results from the simple correlations. Mothers who perceived one of their twins to be ‘fussier’ than the other were more likely to differ in both their likelihood to use food as a reward (‘instrumental feeding’) and the amount of pressure exerted on each twin to eat (Table 3). As the difference in fussiness increased between the twins, the more the mother would differentially pressure or instrumentally feed the twins.

**Table 3:** Models for within-twin pair differences in ‘food fussiness’ and maternal feeding practices (*n* = 1013 pairs)

Within-twin pair differences	Model 1: Instrumental feeding^b^ F(7, 1005) = 4.34 (*p* < .001) R^2^ = .029			Model 2: Pressure to eat^c^ F(7, 1005) = 45.61 (*p* < .001) R^2^ = .241			Model 3: Restriction^d^ F(7, 1005) = 4.39 (*p* < .001) R^2^ = .030		
	B ± SE	β	*p* value	B ± SE	β	*p* value	B ± SE	β	*p* value
Food fussiness^a^	.021 (.004)***	**.146**	**<.001**	.148 (.012)***	**.338**	**<.001**	-.011 (.013)	−.026	.405
SDS Birth weight	.004 (.003)	.046	.203	.005 (.008)	.022	.959	.004 (.008)	.018	.624
SDS Weight at 16 months	−.006 (.003)	−.072	.051	−.092 (.009)***	**−.347**	**<.001**	.038 (.009)***	**.153**	**<.001**
Sex^e^									
Both boys	−.008 (.006)	−.045	.216	.012 (.017)	.021	.505	.029 (.019)	.056	.121
Both girls	−.002 (.006)	−.010	.787	.017 (.017)	.032	.317	.008 (.018)	.017	.643
Early feeding method^f^									
Both mostly breastfed	−.001 (.015)	−.009	.924	−.022 (.042)	−.043	.604	−.009 (.045)	−.018	.845
Both mostly bottle−fed	.006 (.015)	.037	.694	−.009 (.042)	−.019	.823	−.015 (.045)	−.032	.729

*B* indicates unstandardized estimate, *β* indicates the standardized estimate

*p < .05; *p < .01; **p < .001

Subscales are from the ^a^CEBQ [25]; ^b^PFSQ [23]; ^c^CFQ [12]; and ^d^Poppets scale [24]

^e^The reference group different sex between twins (ie. boy-girl twin pairs); ^f^The reference group is different feeding method between twins (ie. one twin mostly bottle-fed, the other twin mostly breastfed)

### Twins discordant in food fussiness

There were 274 twin pairs who were discordant for ‘food fussiness’ (‘food fussiness’ difference score ≥ .50). Means and standard deviations for ‘food fussiness’ and feeding practices for the fussy twin versus the less fussy twin are presented in Table 4. Paired Samples T-tests indicated that, as expected, the fussy and less fussy twin were significantly different on the ‘food fussiness’ scale (t(273) = −31.92, *p* < .001). The results of the subsample supported the results from the larger within-family analyses, showing differences in the use of pressure and instrumental feeding between fussier and less fussy twins. Mothers were significantly more likely to pressure the fussier twin to eat more than the less fussy twin (t(273) = −6.70, *p* < .001). Mothers also reported rewarding their fussier twin child for eating in comparison to the less fussy twin (t(273) = −2.58, *p* = .010). However, the effect sizes were small for both ‘instrumental feeding’ and ‘pressure to eat’ (.02 and .18 respectively). There were no differences in ‘restriction’ between discordant twins (*p* = .176).

**Table 4:** Differences in ‘food fussiness’ and maternal feeding practices for twins discordant in ‘food fussiness’ (*n* = 247)

	Fussy twin *n* = 247	Less fussy twin *n* = 247	*p* value
	Mean (SD)		
Food Fussiness^a^ (range 1–5)	**2.95 (.68)**	**2.00 (.60)**	**<.001**
Instrumental feeding^b^ (range 1–5)	**1.31 (.49)**	**1.30 (.46)**	**.010**
Pressure to eat^c^ (range 1–5)	**2.37 (.74)**	**2.23 (.69)**	**<.001**
Restriction^d^ (range 1–7)	5.12 (1.26)	5.13 (1.26)	.176

Subscales are from the ^a^CEBQ [25]; ^b^PFSQ [23]; ^c^CFQ [12]; and ^d^Poppets scale [24]

### Between-family analyses

The findings from the between-family analyses were slightly different to the within-family analyses insofar as ‘food fussiness’ was significantly associated with all three maternal feeding practices, including ‘restriction’ (Table 5). In keeping with the within-family analyses, ‘food fussiness’ was positively associated with both ‘pressure to eat’ and ‘instrumental feeding’, but it was also significantly negatively associated with ‘restriction’, after controlling for potential confounders (*p* < .001). This indicated that mothers with fussier children exerted more pressure, were more likely to use their favorite foods as a reward, and exerted less restriction over access to, and portion sizes of foods high in sugar and fat.

**Table 5:** Models of between-family analyses for ‘food fussiness’ and feeding practices at 16-months old (*n* = 2026 children)

	Model 1: Instrumental feeding^b^ Wald F(12, 1001) = 6.805 (*P* < .001) R^2^ = .086		Model 2: Pressure to eat^c^ Wald F(12, 1001) = 12.826 (*P* < .001) R^2^ = .139		Model 3: Restriction^d^ Wald F(12, 1001) = 6.385 (*P* < .001) R^2^ = .076	
	B ± SE	*p* value	B ± SE	*p* value	B ± SE	*p* value
Food Fussiness^a^	.081 (.019)***	**<.001**	.289 (.031)***	**<.001**	−.323 (.055)***	**<.001**
Sex of child (female)	.014 (.023)	.556	−.096 (.035)**	**.007**	−.036 (.063)	.572
Education Qualification^ - Low - Medium	.197 (.044)*** .083 (.031)**	**<.001** **.007**	.082 (.063) −.017 (.049)	.196 .722	.019 (.112) .045 (.085)	.867 .597
Ethnicity - Non-white British	.054 (.049)	.276	.278 (.075)***	**<.001**	.168 (.125)	.180
BMI of mother (kg/m^2^)	−.002 (.003)	.525	−.005 (.005)	.385	−.025 (.009)**	**.005**
Age of mother at birth	−.018 (.003)***	**<.001**	−.014 (.004)**	**.001**	.010 (.008)	.217
Age of child (months)	.007 (.015)	.660	−.028 (.023)	.229	.070 (.040)	.082
Gestational age (weeks)	.003 (.005)	.580	−.025 (.008)**	**.003**	.018 (.017)	.275
SDS Birth weight	−.016 (.014)	.278	−.006 (.021)	.783	−.058 (.040)	.146
SDS Weight at 16 months	−.003 (.013)	.817	−.083 (.021)***	**<.001**	.201 (.040)***	**<.001**
Mostly bottle-fed	−.018 (.029)	.538	−.002 (.044)	.962	−.109 (.077)	.157

*B* indicates unstandardized estimate

^^^Reference group is “high” maternal educational qualification (University educated)

*p < .05; **p < .01; ***p < .001

Subscales are from the ^a^CEBQ [25]; ^b^PFSQ [23]; ^c^CFQ [12]; and ^d^Poppets scale [24]

## Discussion

This was the first study to use a twin design to explore if mothers vary their feeding practices for twin children who differ in their fussy eating. As hypothesized, mothers exerted differential levels of ‘pressure to eat’ and reported differential use of food as a reward when one twin was fussier than the other. In a subset of twins who were the most discordant (highest quartile) in fussy eating, (‘food fussiness’ difference score ≥ .50), the results were the same. Mothers reported more ‘pressure to eat’ and ‘instrumental feeding’ when feeding the fussier twin, although differences in parental feeding practice scale scores were small. However, the feeding scales used in this study were not designed as a clinical tool, therefore it is difficult to determine the clinical significance of these differences. Using this novel twin design approach, we demonstrated that mothers of twins who differ in their ‘food fussiness’ may use some different feeding practices with each child. These findings can be interpreted in terms of a child-responsive model of the parent–child feeding relationship and may indicate that parents adopt specific feeding practices in response to their child’s fussy eating tendencies, even in the early toddler period.

The between-family analyses differed slightly from the within-in family analyses insofar as fussy eating was also significantly negatively associated with ‘restriction’, such that fussier children were less restricted for foods high in sugar and fat. The null finding in the within-family analysis for ‘restriction’ may reflect the low within-family variation for this particular parental feeding style, which indicates that ‘restriction’ may be more of a global parental policy than a child-responsive strategy to manage the child’s emerging appetitive traits.

A considerable body of evidence supports the positive cross-sectional association between fussy eating and pressuring feeding practices [10, 27, 28], and findings from this study indicate that this relationship is present from a young age. Parents may intuitively pressure their child to eat to encourage food intake and weight gain, although prospective and experimental studies suggest that pressure may be associated with poorer food intake [8], lack of interest in food [9], and lower weight gain [14]. Nevertheless, it isn’t clear that pressuring a child to eat directly worsens fussy eating behaviors and some degree of “pressure” may serve to mitigate fussy eating difficulties, especially in the short term [29]. Irrespective of the efficacy of this parent feeding strategy, findings from the current study demonstrate that mothers may attempt to manage fussy eating behaviors by pressuring children to eat.

The positive association between ‘food fussiness’ and ‘instrumental feeding’ is in line with previous studies of mothers with fussy children who reported using favored foods as rewards for eating healthy foods or for behaving well [30]. While rewarding a child for eating a less preferred food with a preferred food may increase the child’s intake of the less preferred food in the short term (i.e. for that particular eating occasion), employing a means-end strategy has been shown to negatively shift a child’s preference for the target food in the longer term [11]. Parents appear to employ a variety of techniques to counter food rejection, and the current study suggests that mothers may respond to differences in fussy eating between twin siblings by using a greater level of food rewards with the fussier child. However, relatively few mothers (*n* = 30) reported different levels of ‘instrumental feeding’ for each twin. Differential maternal use of ‘instrumental feeding’ between twins discordant for ‘food fussiness’ is likely to be subtle (mean difference = .01), and as previously mentioned, the clinical significance of this finding is questionable. In comparison to ‘pressure to eat’, ‘instrumental feeding’ seems less influenced by child behaviour.

The inverse association between fussy eating and restriction of high fat or high sugar foods in the between-family analyses contrast with findings from previous studies. The majority of cross-sectional studies in older children have reported that mothers of fussy eaters were more likely to restrict their child’s food intake [9, 10, 31, 32]. Previous studies have speculated that parents may restrict fussy eater’s food intake for health reasons [32], and perhaps believe that withholding palatable, energy-dense foods from the child may encourage consumption of rejected foods, such as fruit and vegetables [18]. It has been hypothesized that restriction could increase preference for the restricted food [33], potentially exacerbating fussy eating, however this hypothesis is yet to be confirmed.

Factors that may account for the difference between previous findings and ours include the young age of our sample and the use of a different tool to measure restriction. Most past studies used the ‘restriction’ subscale from the CFQ which asks about restriction of the child’s “favorite foods” (which does not necessarily mean foods high in sugar or fat); does not ask about restriction of portion sizes, and does not discriminate between limiting a child’s access to food and using food as a reward or in exchange for good behavior. There was very little difference in the use of restriction within families, even when twins were discordant in ‘food fussiness’. This suggests that restriction reflects a more general feeding practice rather than a child responsive strategy, and it may indicate that mothers who are less restrictive encourage greater fussiness in their children because they become accustomed to enjoying unlimited access to foods high in sugar and fat, and are therefore more likely to reject less palatable foods such as vegetables. It may also be the case that it is more difficult to vary levels of restriction with two twins than it is to use differing levels of pressure or food as reward. Qualitative research may help to better understand the relationship between ‘restriction’ and fussy eating in toddlerhood.

Few studies have examined longitudinal relationships between parental feeding practices and children’s fussy eating behavior. Indeed, to the authors’ knowledge, prospective studies have only examined whether particular feeding practices (mainly ‘pressure to eat’ or ‘restriction’) are associated with greater subsequent food fussiness [1, 9] or lower child weight [34], rather than the reverse direction. In a recent Australian study of parents of 2- to 5- year old children, associations between feeding practices (‘restriction’ and ‘pressure to eat’) and ‘food fussiness’ one year later were found to attenuate when controlling for additional covariates, for example, child temperament [35]. The NOURISH RCT found that in comparison to ‘usual care’, anticipatory guidance on early feeding improved responsive feeding practices in mothers [36] and resulted in improved dietary outcomes, food preferences and eating behaviors in children 3.5 years after the intervention [37]. However, no differences were observed in children’s ‘food fussiness’ between the control and intervention group, suggesting that feeding practices may not be able to influence fussy eating behaviors. Longitudinal analysis of the potentially bidirectional effects between ‘food fussiness’ and parental feeding practices are needed to fully explore the complexities of the relationship and to understand how these may change over the course of development.

### Strengths, limitations and future directions

Children in this study were very young (16-months old), allowing us to capture fussy eating as it emerged. The twin design allowed us to control for a number of potentially confounding factors pertaining to aspects of the early environment shared by twin pairs, providing stronger evidence for the idea that mothers adapt their feeding practices to each child’s emerging eating behaviors, in a child-responsive framework. Lastly, the sample was large, and therefore reliable associations between parental feeding practices and child ‘food fussiness’ were established, while adjusting for a range of individual-level covariates including child weight. Consequently, the findings suggest that parents adapt their feeding practices to accommodate their child’s fussiness, independently of their weight.

Twins are born earlier and smaller than singletons, and there may be more concern about early growth while twin children “catch up”. Parents may be more likely to pressure and less likely to restrict their twin children compared to singletons. However, there was variation in the fussiness (and weight) of children, and weight and gestational age were controlled for in the analysis. There is no reason to believe that the relationships between parental feeding and fussiness would be different in a twin sample. The scales used in the current study were adapted for the younger age of the children; but these scales need to be formally validated in toddlers. The internal reliability for the ‘pressure to eat’ and ‘instrumental feeding’ scales were low and did not reach the recommended Cronbach’s Alpha value of 0.70 [38]. While the within-family design may support a child-responsive feeding model, the data in this study were nevertheless cross-sectional and thus directionality cannot be determined. A longitudinal within-family study investigating changes in both ‘food fussiness’ and parental feeding practices from toddlerhood to early childhood would provide a greater understanding of directionality.

## Conclusion

This was the first study to use a twin design to explore fussy eating and feeding practices, within-families. Results from this study suggest that mothers used more pressure and instrumental feeding practices with their fussier twin than with his or her co-twin. These findings provide support for the idea that feeding practices that aim to increase a child’s consumption of a particular type or quantity of food may develop in response to a child’s emerging fussy eating behavior.

## Abbreviations

CFQ, child feeding questionnaire; PFSQ, parental feeding style questionnaire; BMI, body mass index CEBQ, children’s eating behaviour questionnaire; SDS, standard deviation score

## Additional file

Additional file 1: Parental feeding practices subscales and items used in the Gemini questionnaire when children were 16 months old. (DOC 23 kb)
