# Supplementary material for: Maternal feeding practices and fussy eating in toddlerhood: a discordant twin analysis
Source: Int J Behav Nutr Phys Act. 2016 Jul 13;13:81. doi: 10.1186/s12966-016-0408-4 (PMC4944306; doi:10.1186/s12966-016-0408-4)
Supplement: Additional file 1: — Parental feeding practices subscales and items used in the Gemini questionnaire when children were 16 months old. (DOC 23 kb) [file 12966_2016_408_MOESM1_ESM.doc]

**Additional file 1:** Parental feeding practices subscales and items used in the Gemini questionnaire when children were 16 months old

Instrumental Feeding

1. If my child misbehaves I withhold his/her favorite food
2. I use puddings as a bribe to get my child to eat his/her main course
3. I reward my child with something to eat when s/he is well-behaved

Pressure to eat

1. My child should always eat all of the food I give him/her
2. I have to be especially careful to make sure my child eats enough
3. If my child thinks he/she isn’t hungry, I try to get him/her to eat anyway
4. If I did not guide or regulate my child’s eating, s/he would eat much less than s/he should
5. I insist my child eat some fruit or vegetables, even if s/he doesn’t want

Restriction

1. I limit my child’s access to sugary foods
2. I limit my child’s access to high fat foods
3. I limit the portion sizes of high fat foods that I give to my child
4. I limit the portion sizes of sugary foods that I give to my child
